# 41‐year‐old male with a pituitary mass

**DOI:** 10.1111/bpa.12961

**Published:** 2021-07-19

**Authors:** Wenjuan Wen, Leiming Wang, Mengyou Li, Peijin Li, Yubo Ren, Xuedong Zhang

**Affiliations:** ^1^ Department of Pathology Liaocheng People's Hospital Liaocheng China; ^2^ Department of Pathology Xuanwu Hospital Capital Medical University Beijing China; ^3^ Department of Neurosurgery Liaocheng People's Hospital Liaocheng China

## Abstract

Cranial coronal T1‐weighted magnetic resonance imaging with contrast enhancement showed a sellar irregular lesion (Figure A). Hematoxylin and eosin staining showed two different morphologies. The majority of tumor cells had medium‐sized to large cells with a high nucleus to cytoplasm ratio, vesicular nuclei with prominent nucleoli, and poor adhesion (Figure B), which revealed positive expression of CD20 by Immunohistochemistry (Figure C). The other component showed abundant cytoplasm, spindle‐like to ovoid nucleus and rare mitotic figures (Figure D). These tumor cells were positive for Pit‐1 (Figure E) and perinuclear punctated structures immunopositive for CK18 (Figure F).

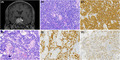

BOX 1 **Slide scan**

Access the whole slide scan at http://image.upmc.edu:8080/NeuroPathology/BPA/BPA‐20‐11‐293.svs/view.apml

## 1 CLINICAL HISTORY AND IMAGING

**FIGURE 1:**
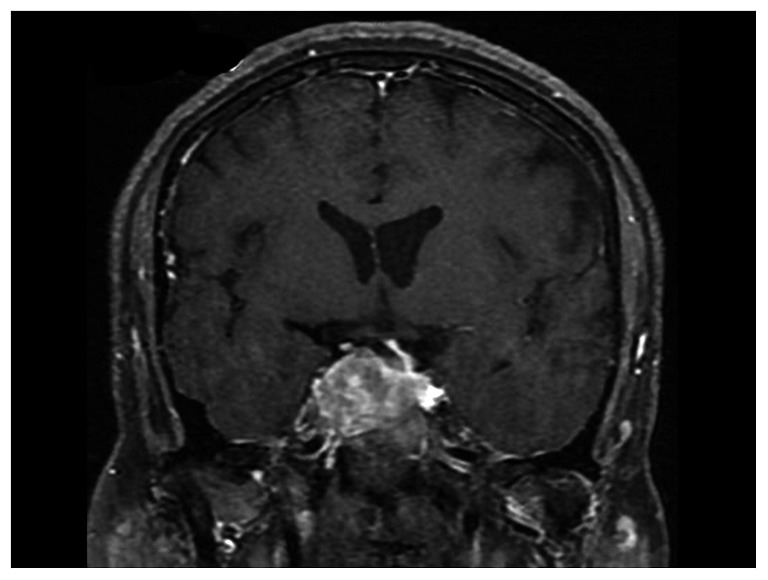
Cranial coronal T1‐weighted MRI with contrast enhancement showed a sellar irregular lesion with a leftward deflection of the hypophysis stalk

A 41‐year‐old male developed an intermittent stabbing headache without clear inducement 8 days. The headache was widespread and accompanied by fatigue, which could be relieved to some extent after rest. The patient had no other obvious symptoms. Cranial MRI (magnetic resonance imaging) revealed an intrasellar lesion with depressed sellar floor. The lesion showed hypointense T1‐weighted and heterogeneously isointense T2‐weighted image signals with heterogeneous contrast enhancement. It has unclear boundaries with clivus, and the hypophysis stalk showed a leftward deflection (Figure 1). These findings suggested a pituitary macroadenoma. The serum level of ACTH and TSH was mildly elevated to 94.0 pg/ml (normally 10.1–57.6 pg/ml) and 6.84 mIU/L (normally 0.465–4.680 mIU/L) respectively, with normal GH, PRL, FSH and LH levels. Serology for human immunodeficiency virus was negative. The patient underwent neuroendoscopic tumor resection via the nasal sinus.

## 2 MICROSCOPIC PATHOLOGY AND DIAGNOSIS

**FIGURE 2:**
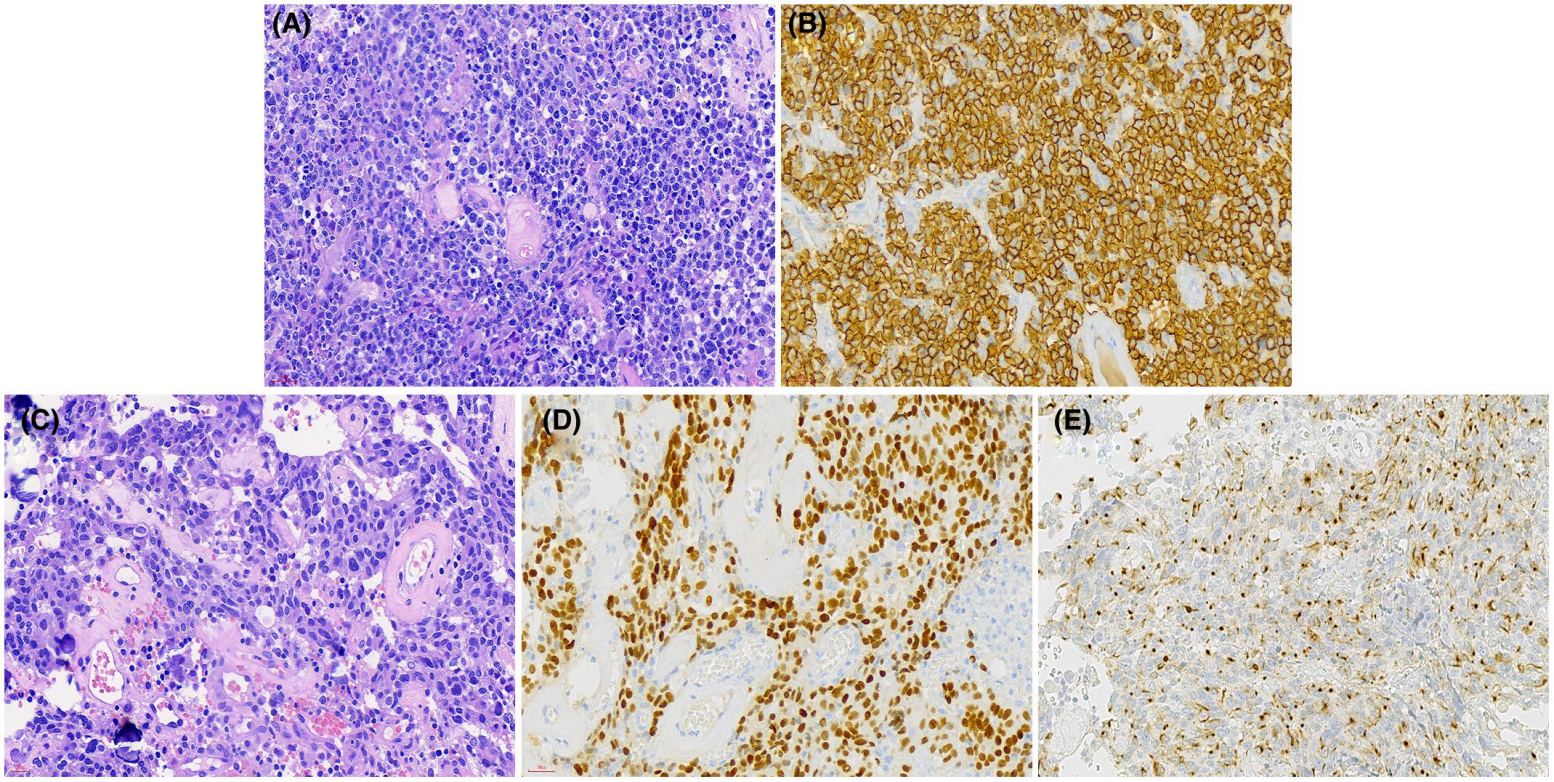
HE staining showed two different morphologies. The majority of tumor cells had medium‐sized to large cells with a high nucleus to cytoplasm ratio, vesicular nuclei with prominent nucleoli, and poor adhesion (A), which revealed positive expression of CD20 by Immunohistochemistry (B). The other component showed abundant cytoplasm, spindle‐like to the ovoid nucleus and rare mitotic figures (C). These tumor cells were positive for Pit‐1 (D) and perinuclear punctated structures immunopositive for CK18 (E). H&E, hematoxylin and eosin; MRI, magnetic resonance imaging

Microscopic examination of the sample exhibited two different morphologies by HE staining. Most of the tumor tissues showed poor adhesion, significant cytological atypia, large cells with a high nucleus to cytoplasm ratio, vesicular nuclei with prominent nucleoli, and mitoses are frequent (Figure 2A). Immunohistochemical analysis of this region revealed positive expression of CD45, CD20 (Figure 2B), CD79α, CD10, Bcl‐6 and c‐MYC (15%+), partial expression of MUM1. Bcl‐2 was negative. The Ki‐67 labeling index was approximately 80%. MYC rearrangements were not detected by split‐signal FISH analysis. Another tumor showed distinct morphological profiles, including abundant cytoplasm, spindle‐like to ovoid nucleus and rare mitotic figures (Figure 2C). These tumor cells were positive for Pit‐1 (Figure 2D), PRL and Syn, with perinuclear punctated structures immunopositive for cytokeratin 18 (CK18) (Figure 2E). The ki67 labeling index was <2%. The expression of SF‐1, T‐Pit, GH, TSH, FSH, LH, and ACTH was negative. One month after surgery, positron‐emission tomography‐CT (PET‐CT) imaging of the whole body demonstrated no suspicious lesions in other locations. Bone marrow biopsy and cerebrospinal fluid (CSF) analysis excluded disseminated disease.

## 3 DIAGNOSIS

Primary pituitary diffuse large B‐cell lymphoma, combined with sparsely granulated lactotroph adenoma.

## 4 DISCUSSION

Primary pituitary lymphoma of the sellar region, defined as a lymphoma confined to the pituitary gland and sellar region at presentation, is extremely rare. The literature to date includes <40 cases of primary lymphoma in the sellar region in patients with normal immune function, primarily B‐cell lymphomas (82%) and mostly diffuse large B‐cell (63%) (1), In six cases the lymphoma occurred in association with pituitary adenoma, in 4 patients concomitant like in our, while in 2 the diagnosis of adenoma preceded that of lymphoma by 17 and 25 years respectively. Contrasting to previously reported cases with an average age of 68.8, our patient is younger. To the best of our knowledge, a combination of lymphoma with FSH, TSH, GH and ACTH adenoma has previously been reported in the literature, but not with PRL producing adenoma (2, 3).

There are different theories regarding the possible mechanism underlying the development of primary sellar lymphoma. The malignant transformation of resident lymphocytes or those that enter the central nervous system due to inflammation, lymphocytic hypophysitis and pituitary adenoma are commonly considered to be risk factors. The prolactin receptor is expressed on many types of immune cells, and prolactin is secreted by adenoma cells which are known to have mitogenic effects on lymphocytes. When lymphoma in the sellar region involves the hypothalamus or the stalk of the hypophysis, it can disconnect the dopamine secretion pathway, which weakens the inhibitory effect of dopamine on the prolactin‐secreting cells of the hypophysis. This in turn leads to prolactin secretion and abnormal growth, finally leading to the development of prolactinoma.

Due to the limited number of cases and lack of specific research, most of the treatments are based on the recommendations for primary central nervous system lymphoma. High‐dose methotrexate is the basis of many systemic chemotherapy regiments, and has greatly improved the survival rates. In the present case, R‐MI chemotherapy regimen was administered for three courses, including high‐dose Methotrexate, rituximab, and ifosfamide with intrathecal injection and systemic chemotherapies. There have been no recurrence 9 months by imaging examinations after surgery, and the patient was asymptomatic. Because of the unusual anatomical location of the sellar region exempt from the blood‐brain barrier, primary sellar region lymphoma should be differentiated from lymphoma occurring in other areas of the central nervous system.

## DATA AVAILABILITY STATEMENT

Data available on request from the authors.
